# Expansion of the clinical and neuroimaging spectrum associated with NDUFS8‐related disorder

**DOI:** 10.1002/jmd2.12303

**Published:** 2022-08-23

**Authors:** Milena M. Andzelm, Shanti Balasubramaniam, Edward Yang, Alison G. Compton, Kate Millington, Jia Zhu, Irina Anselm, Lance H. Rodan, David R. Thorburn, John Christodoulou, Siddharth Srivastava

**Affiliations:** ^1^ Department of Neurology Children's Hospital Boston Boston Massachusetts USA; ^2^ Western Sydney Genetics Program The Children's Hospital at Westmead Sydney New South Wales Australia; ^3^ Department of Metabolic Medicine and Rheumatology Perth Children's Hospital Perth Western Australia Australia; ^4^ Department of Radiology Children's Hospital Boston Boston Massachusetts USA; ^5^ Murdoch Children's Research Institute Melbourne Victoria Australia; ^6^ Department of Paediatrics University of Melbourne Melbourne Victoria Australia; ^7^ Division of Endocrinology, Department of Pediatrics Children's Hospital Boston Boston Massachusetts USA; ^8^ Division of Genetics and Genomics, Manton Center for Orphan Disease Research, Department of Pediatrics Children's Hospital Boston Boston Massachusetts USA; ^9^ Victorian Clinical Genetic Services Melbourne Victoria Australia

**Keywords:** autoimmune diabetes, mitochondrial disorder, NDUFS8, progressive leukodystrophy

## Abstract

Biallelic pathogenic variants in *NDUFS8*, a nuclear gene encoding a subunit of mitochondrial complex I, result in a mitochondrial disorder characterized by varying clinical presentations and severity. Here, we expand the neuroimaging and clinical spectrum of NDUFS8‐related disorder. We present three cases from two unrelated families (a girl and two brothers) homozygous for a recurrent pathogenic *NDUFS8* variant [c.460G>A, p.(Gly154Ser)], located in the [4Fe‐4S] domain of the protein. One of the patients developed auto‐antibody positive diabetic ketoacidosis. Brain MRIs performed in two of the three patients demonstrated diffuse cerebral and cerebellar white matter involvement including corticospinal tracts, but notably had sparing of deep gray matter structures. Our report expands the neuroimaging phenotype of NDUFS8‐related disorder to include progressive leukodystrophy with increasing brainstem and cerebellar involvement, with relative sparing of the basal ganglia. In addition, we describe autoimmune diabetes in association with NDUFS8‐related disorder, though the exact mechanism of this association is unclear. This paper provides a comprehensive review of case presentation and progressive neuroimaging findings of three patients from two unrelated families that have an identical pathogenic *NDUFS8* variant, which expands the clinical spectrum of NDUFS8‐associated neurological disease.

**Synopsis**

This paper provides a comprehensive review of case presentation and progressive neuroimaging findings of three patients from two unrelated families that have an identical pathogenic *NDUFS8* variant, which expands the clinical spectrum of NDUFS8‐associated neurological disease.

## 1 INTRODUCTION

*NDUFS8 (NADH:Ubiquinone Oxidoreductase Core Subunit S8)* is a nuclear gene that encodes a core subunit of mitochondrial complex I (CI), an enzyme complex that catalyzes the first step in the electron transport chain resulting in the generation of energy in the form of ATP. NDUFS8 is a highly conserved protein with an iron–sulfur [4Fe‐4S] binding site, which facilitates electron transfer to ubiquinone as part of the Q module of CI.^1^

In humans, biallelic pathogenic variants in *NDUFS8* often underlie severe presentations of mitochondrial disease, including Leigh syndrome and mitochondrial encephalopathy (MIM# 602141).^2^, ^3^ NDUFS8‐related disorder was first described in an infant with Leigh syndrome (LS) and cardiomyopathy who died at 11 weeks of age.^4^ Since then, several other patients have been described with severe manifestations, often including cardiomyopathy, as well as epilepsy, severe developmental delays and imaging abnormalities often involving the white matter, basal ganglia and brainstem.^4^, ^5^, ^6^

Recent studies have demonstrated milder clinical phenotypes associated with NDUFS8‐related disorder. These patients generally presented in childhood, and MRI brain demonstrated signal change in the putamen.^7^, ^8^ Their symptoms were slowly progressive over years with variable manifestations of Leigh syndrome. For example, one study described a 9‐year‐old girl with late‐onset LS manifesting as nystagmus, dysarthria, dystonia, involuntary hand movements, toe walking, and ataxic gait.^7^ Another study described a family with three children with Leigh syndrome, still alive at 9–13 years old, presenting with variable features of developmental delay, dysarthria, progressive external ophthalmoplegia, ptosis, muscle weakness, and ataxia.^8^

Given that the total number of individuals reported with NDUFS8‐related disorder is small, the overall knowledge about this disorder is limited. In this report, we present three affected individuals from two unrelated families (a girl and two brothers) with a recurrent homozygous pathogenic variant in *NDUFS8*, c.460G>A; p.(Gly154Ser). The two brothers, but not the girl, have been previously reported, though with limited clinical information.^6^ All three individuals developed regression and died after subsequent regressive setbacks in the setting of illness. The girl acutely decompensated in association with an upper respiratory viral infection and diabetic ketoacidosis (DKA) in the setting of new autoimmune diabetes.

## 2 KEY POINTS

The significance of this report is as follows:

1. We have provided comprehensive review of the neuroimaging findings of the patients, which is much needed given that brain MRI can be a first step toward this molecular diagnosis.
2. We have expanded the clinical spectrum of NDUFS8‐associated neurological disease to include progressive leukodystrophy with increasing brainstem and cerebellar involvement, with relative sparing of the basal ganglia.
3. We describe autoimmune diabetes in association with NDUFS8‐related disorder, though the mechanism of this association is unclear.

## 3 CLINICAL REPORT

See Table 1 for summary of clinical details.

**TABLE 1:** Clinical characteristics of individuals in this cohort

	Patient 1	Patient 2	Patient 3 (sibling of patient 2)
Genetics			
Variant	c.460G>A, p.(Gly154Ser), homozygous, heterozygous in parents	c.460G>A, p.(Gly154Ser), homozygous in both affected siblings, heterozygous in parents	c.460G>A, p.(Gly154Ser), homozygous in both affected siblings, heterozygous in parents
Consanguinity	No	No	No
Demographics			
Ethnicity	Indian	Sudanese	Sudanese
Sex	Female	Male	Male
Age at last exam	24 months	14 months	14 months
Deceased	Yes (24 months)	Yes (14 months)	Yes (15 months)
Cause of death	Bradycardic arrests in setting of metapneumovirus virus infection	Parainfluenza pneumonia	Uncontrolled seizures and respiratory compromise
Perinatal history			
Gestational age	36 weeks	41 weeks	39 weeks
Birth weight	1.81 kg	3.54 kg	3.28 kg
Systemic features			
Cardiomyopathy	Y	Not known	Not known
Diabetes mellitus	Y	Not known	Not known
Development			
Regression	Yes	Yes	Yes
Onset of regression	9 months (after viral illness)	6 months (after febrile illness)	5 months
Nature of regression	Lost ability to sit independently, crawl, or pull to stand	Lost ability to sit with support, roll over, bear weight on legs, and reach for objects.	Lost musical vocalizations
Language abilities	18 months: could smile socially, laugh out loud, babble, say mama nonspecifically	7 months: could smile socially, mouth objects	5 months: could make musical vocalizations
Motor abilities	18 months: could lift head up in prone	7 months: poor head control, unable to roll over	5 months: could roll to side but not supine to prone or prone to supine; in prone unable to lift head or chest against gravity
Neurological features			
Seizures	N	N	Y
Axial hypotonia	Y	Y	Y
Appendicular spasticity	Y	Y	Y
Dystonia	Y	N	N
Hyperreflexia	Y	Y	Y
MRI features			
Age of latest scan	24 months	7 months	Not done
Involvement of diffuse cerebral white matter	Y	Y	
Involvement of corticospinal tracts	Y	Y	
Involvement of middle cerebellar peduncles	Y	N	
Involvement of cerebellar white matter	Y	Y	
Diffusion restriction in affected areas	Y (10 months: more diffuse; 24 months: receded to subcortical U fibers)	Y	
Enhancement	Y	N	
Elevated lactate on MRS	Y (at 24 months), N (at 10 months)	Y (7 months)	
Sparing of basal ganglia	Y	Y^a^	
Prior studies			
Plasma lactate	1.8 mmol/L (ref range 0.5–2)	3.0–3.6 mmol/L (ref range 0.0–2.0)	2.6 mmol/L (ref range 0.0–2.0)

^a^ For patient 2, basal ganglia spared on initial imaging, though were involved on histopathological analysis on autopsy 7 months later.

### 3.1 Patient 1

Patient 1 was born at 36 weeks gestation with a birth weight of 4 pounds (small for gestational age). Early development was notable for typical acquisition of motor milestones until motor regression at 8 months of age. She crawled and rolled at 6 months of age, and she sat unsupported at 7 months of age. However, at 8 months of age, a few days after a viral illness, she lost the ability to roll over, crawl, sit independently, and reach for objects. She developed irritability and sleeping difficulty.

Family history was notable for the father having a history of IgA nephropathy and kidney transplantation, and the paternal grandfather also had a history of kidney transplantation. Family history was otherwise negative for neurodevelopmental or other autoimmune disorders. There was no known consanguinity.

On her neurological exam at 10 months, she appeared irritable. She had axial hypotonia, appendicular spasticity (lower > upper) with admixed dystonia. She could not sit independently, reach for objects, or transfer objects from one hand to another. Reflexes were 3+ with upgoing toes bilaterally.

She developed failure to thrive requiring eventually G‐tube placement. Echocardiogram demonstrated concern for hypertrophic cardiomyopathy. At the age of 24 months, she presented with DKA secondary to new‐onset diabetes mellitus in the setting of metapneumovirus infection. Her pancreatic autoantibodies (anti‐GAD, anti‐IA2, anti‐insulin, anti‐Zn62) were pan‐positive. Despite treatment and resolution of her DKA, she died after developing episodes of bradycardic arrests thought to be secondary to progressive brainstem dysfunction.

Initial metabolic investigations included urine organic acids with trace quantities of 3‐hydroxyisobutyric, ethylmalonic, fumaric and malic acids, plasma very long chain fatty acids with ratio of C24/C22 and C26/C22 slightly higher than normal but normal amount of C26:0. Plasma amino acids with elevated alanine but obtained with tourniquet. Blood ammonia and white blood cell lysosomal enzyme activity were normal. Blood lactate was 1.8 mmol/L (normal range 0.5–2.0), pyruvate was not obtained. CSF metabolic studies were not obtained.

**FIGURE 1:**
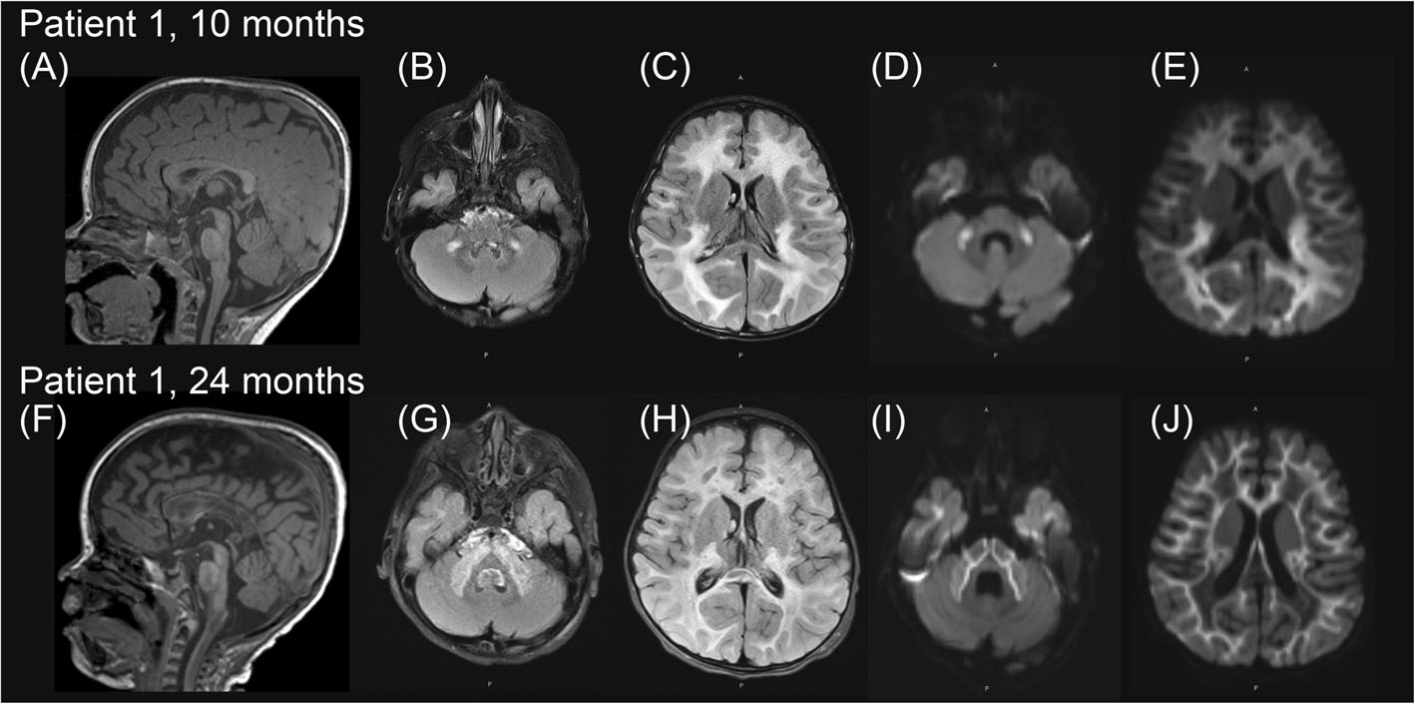
Progressive leukodystrophy in patient 1. Brain MRI of patient 1 at 10 months (top) and 24 months (bottom). Both MRIs performed at 3 Tesla. At 10 months of age, (A) Sagittal T1 MPRAGE and (B and C) axial T2 FLAIR show confluent symmetric T2 prolongation in cerebral white matter (including portions of the corticospinal tracts), with small foci of T2 hyperintensity in the middle cerebellar peduncles and cerebellar white matter. (D and E) axial diffusion weighted imaging shows diffusion restriction in the sites of T2 signal abnormality apart from sparing of areas in the deep white matter which appear mildly expansile. There is sparing of the deep gray matter and brainstem/cerebellar nuclei. There is some enhancement in the posterior periventricular white matter, no detectable lactate on MR spectroscopy performed at 30 and 135 ms echo time (data not shown). By 24 months, (F) sagittal T1 MPRAGE and (G and H) axial T2 FLAIR show new/increased expansile T2 signal abnormality involving the lateral thalami, internal capsules, pons, middle cerebellar peduncles, medullary olives, cerebellar white matter, and cervicomedullary junction. (I and J) axial diffusion weighted imaging shows that diffusion restriction in the cerebral white matter has receded to the subcortical U‐fibers (previously near homogeneously involved the subcortical, deep, and periventricular white matter). There is mild interval loss of cerebral and callosal volume with evidence of some interval demyelination/dysmyelination in the corpus callosum. There is overlapping patchy enhancement and focal lactate accumulation in the thalami (data not shown). MR spectroscopy shows a depressed NAA peak and new lactate accumulation in the evaluable areas of signal abnormality (i.e., thalami) (data not shown).

Initial MRI brain at 10 months of age showed confluent, symmetric areas of T2 prolongation in the cerebral white matter, as well as parts of the middle cerebellar peduncles and cerebellar white matter. There was diffusion restriction in these areas, except for areas in the deep white matter which appeared mildly expansile and diffusion facilitated. By 24 months, this pattern had evolved, showing new/increased expansile T2 signal abnormality involving the lateral thalami, internal capsules, pons, middle cerebellar peduncles, medullary olives, cerebellar white matter, and cervicomedullary junction. At the same time, preexisting areas of cerebral white matter diffusion restriction had resolved apart from the subcortical U‐fibers (previously, there was near homogeneous involvement of the subcortical, deep, and periventricular white matter) and in many locations had undergone cavitation (Figure 1). MR spectroscopy showed a depressed NAA peak and new lactate accumulation.

GeneDx Leukodystrophy Xpanded Panel revealed a homozygous variant in *NDUFS8* (c.460G>A, p.Gly154Ser), with each parent being a heterozygous carrier.

### 3.2 Patient 2

Patient 2 was previously reported,^6^ though clinical details of his presentation in that report are limited. He was the first child of Sudanese parents not known to be consanguineous. The pregnancy was uncomplicated. He was born at 41 weeks gestation by lower segment cesarean section for failure to progress. Apgar scores were 9 and 9 at 1 and 5 min of life, respectively, and there were no perinatal problems. His birthweight, length, and head circumference were within normal range.

He had normal development during the first 6 months of life. However, at 6 months of age, he had an acute neurological deterioration following an intercurrent febrile illness, with loss of previously attained skills, including head control and ability to roll, and slow recovery. At this time, he was centrally hypotonic, peripherally hypertonic, with scissoring of his lower limbs, and exhibited hyperreflexia and fisting of his hands. Weight was 9.2 kg (50th–90th percentile) and head circumference was 46.1 cm (90th percentile).

Over the first 12 months of life, he had several intercurrent respiratory or gastrointestinal illnesses without apparent metabolic decompensation. However, at 14 months of age, he developed a parainfluenza respiratory tract infection and rapidly deteriorated, requiring mechanical ventilation. Despite intensive management over several weeks, there was no improvement. He died after treatment was withdrawn.

Normal metabolic investigations included urine amino acids, urine organic acids, blood ammonia, plasma very long chain fatty acids, white blood cell lysosomal enzyme activities. Blood lactate levels ranged from 3 to 3.6 mmol/L (normal range 0.0–2.0). CSF lactate was marginally elevated at 2.3 mmol/L (normal <2.0) at a time when his blood lactate was 3.0.

**FIGURE 2:**
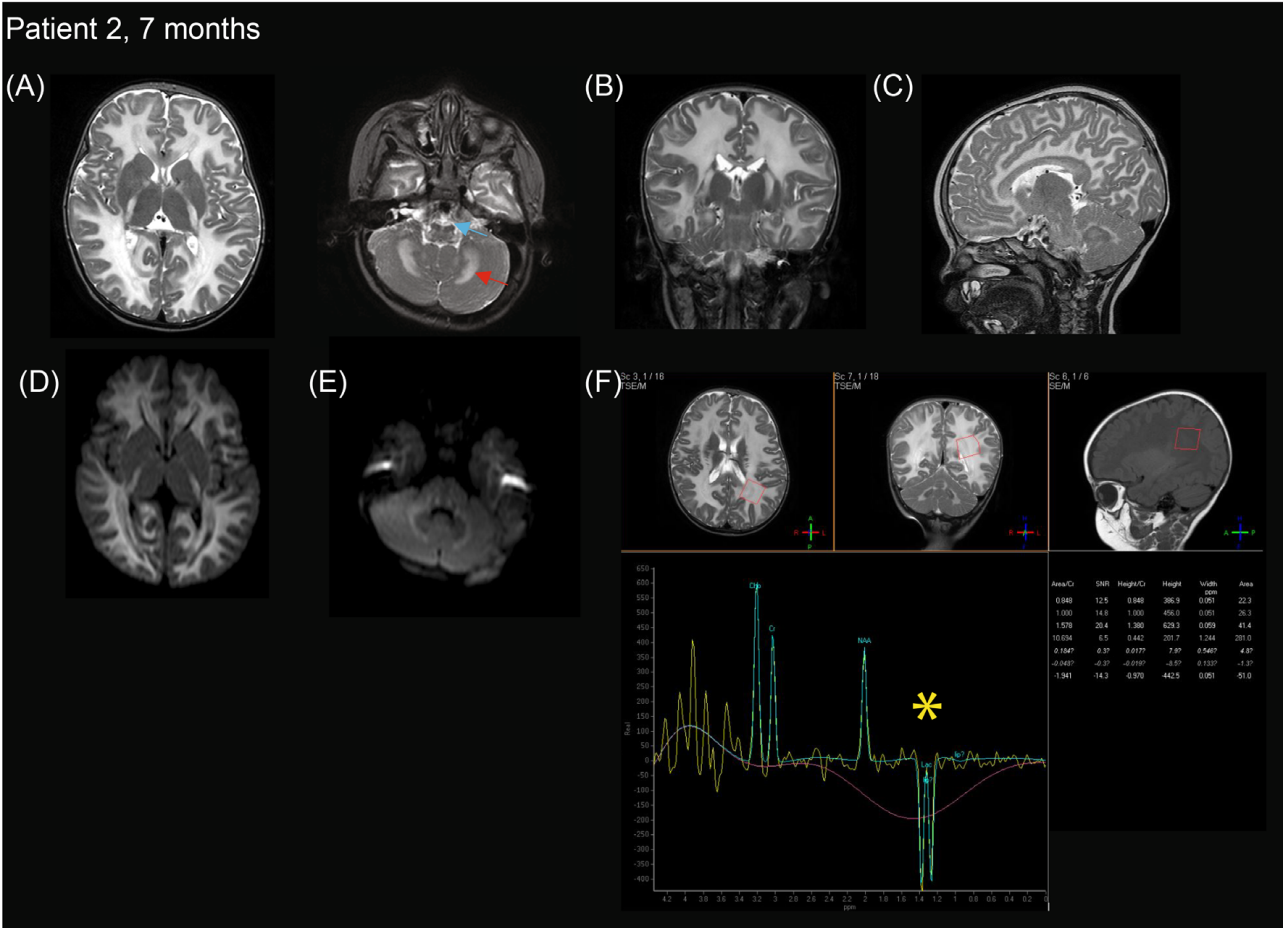
Brain MRI of patient 2 at 7 months. T2 (A) axial (B) coronal and (C) sagittal imaging demonstrate symmetric hyperintensity in supratentorial white matter, corticospinal tracts (blue arrow), and cerebellar white matter (red arrow). Basal ganglia and thalami are relatively spared. Restricted diffusion is seen in a similar distribution to white matter changes in supratentorial white matter (D) and cerebellar white matter (E). (F) MR spectroscopy at 136 ms echo time demonstrates lactate peak (yellow star) and reduced NAA. MRI performed at 1.5 Tesla.

MRI brain at 7 months of age showed involvement of the supratentorial white matter, corticospinal tracts, and cerebellar white matter with restricted diffusion in a similar distribution; MR spectroscopy demonstrated lactate peak (Figure 2). There was no enhancement on post‐contrast imaging.

At autopsy, his brain showed a spongiform leukoencephalopathy involving the cerebrum (with focal involvement of the basal ganglia and thalami), brainstem, and cerebellum, with relative preservation of gray matter and the corpus callosum. Liver histology was normal. Muscle histology showed mild steatosis and occasional fibers with subsarcolemmal accumulation of NADH staining. SDH staining was normal and no COX negative fibers were seen, nor were there any overt ragged red fibers. Lungs showed a severe organizing pneumonia. The kidneys were mostly normal, although there were some sclerosing glomeruli with cellular crescents and focal interstitial inflammation, consistent with a mild focal glomerulonephropathy.

Next‐generation sequencing identified a homozygous variant in *NDUFS8* (c.460G>A, p.Gly154Ser).^6^ Mitochondrial respiratory chain enzymology was performed in liver and muscle collected within 2 h of death. Biochemical testing revealed significantly decreased complex I activity in skeletal muscle (8% of normal control mean) and liver (21% of normal control mean). Activity of other electron transport chain enzymes (complexes II, III, and IV) were in the normal range in skeletal muscle (107%, 82%, and 52%, respectively) and elevated in liver (141%, 175%, and 144%, respectively). All activities were measured as reported elsewhere^9^ and expressed as percent of control mean relative to citrate synthase activity.

### 3.3 Patient 3

Patient 3, the younger sibling of patient 2, was born at term by elective lower segment cesarean section following a pregnancy complicated by gestational diabetes treated with insulin. His Apgar scores were 9 and 9 at 1 and 5 min of life, respectively. Birthweight, length, and head circumference were within normal ranges. There were no problems in the postnatal period.

When evaluated at 5 months of age, he was noted to have developmental delay. At 7 months of age, his weight was 8.43 kg (41st percentile), length was 74 cm (94th percentile), and head circumference was 47 cm (97th percentile). He was unable to sit unaided and was unable to hold his head upright for a prolonged time. Cardiovascular, respiratory, and abdominal examinations were normal. He had mild central and peripheral hypotonia. Deep tendon reflexes were increased.

His urine amino acids and urine organic acid screen was normal, and blood lactate was mildly elevated at 2.6 mmol/L. A vitamin cocktail containing riboflavin, vitamin C, vitamin K, and coenzyme Q was commenced but did not provide clinical benefit. He was subsequently lost to follow up and died at home at age 15 months.

Targeted sanger sequencing confirmed that he had the same *NDUFS8* variant as his brother. Electron transport chain enzyme activities in skin fibroblast mitochondria were below the normal range for complex I (53%) and normal to elevated for complexes II, III, and IV (225%, 171%, and 105%, respectively).

## 4 DISCUSSION

**FIGURE 3:**
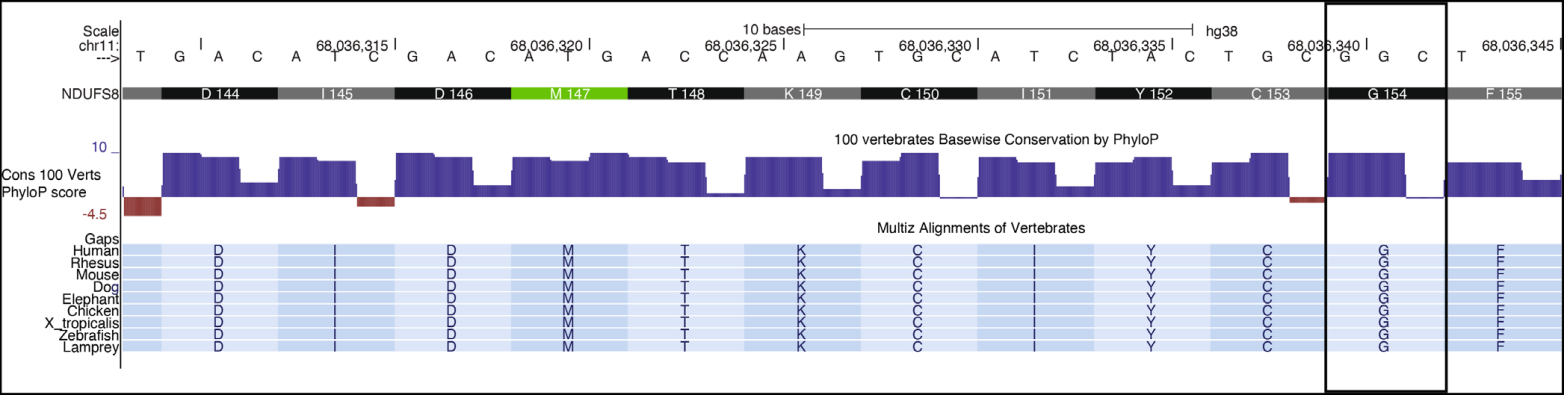
Evolutionary conservation of p.Gly154 residue of *NDUFS8*. Top is cDNA and amino acid sequence. Middle shows 100 vertebrates Basewise Conservation by PhyloP of corresponding residues. Bottom shows Multiz Alignment data from multiple vertebrate species. The box indicates the p.Gly154 residue, which is altered to Serine in our patients. *Source*: Data courtesy of UCSC Genome Browser (http://genome.ucsc.edu)

In this report we have described three patients with the same homozygous variant in *NDUFS8*. There is strong evidence of pathogenicity of the *NDUFS8* c.460G>A (p.Gly154Ser) variant reported in these patients. This variant affects a highly conserved residue of a key functional domain, the Fer4 iron–sulfur cluster binding domain, which is important for electron transport (Figure 3).^10^

In silico prediction tools suggest that this variant is probably damaging (polyphen2 score^11^ 1.000; REVEL score^12^ 0.958; and CADD score^13^ 32.0). This variant results in a non‐conservative amino acid substitution that likely alters polarity within this highly conserved [4FE‐4S] binding site. It is not observed at a significant frequency in large population cohorts (heterozygous in 3 of 183 606 alleles per gnomAD v2.1.1 and v3.1.1 combined).^14^ Finally, CI activity in patients 2 and 3 was significantly reduced in all tissues tested.

The three patients described in this report had severe presentations with death within the first 2 years of life. This degree of disease severity is likely in part attributable to the disruption of this critical [4FE‐4S] binding site.

In addition, patient 1 presented with severe DKA with positive anti‐pancreatic antibodies, suggestive of an autoimmune etiology. The exact relationship between the mitochondrial disorder and the pancreatic autoimmunity in this patient is unclear. Diabetes is a well‐recognized complication of mitochondrial disease, and indeed certain mutations in mitochondrial DNA are well associated with diabetes, for example, maternally inherited diabetes and deafness (MIDD).^15^ However, mitochondrial diabetes is not usually associated with the presence of pancreatic autoantibodies, and HLA‐polymorphisms associated with increased susceptibility to Type 1 diabetes are not associated with the diabetes phenotype in MIDD.^15^, ^16^

Autoimmune or “Type 1‐like” diabetes has only been rarely reported in MELAS syndrome (mitochondrial myopathy, encephalopathy, lactic acidosis and stroke‐like episodes)^17^ and MIDD.^18^, ^19^ Thus, it may be possible that there is coincidental overlap between NDUFS8‐related disorder and autoimmune diabetes in this case. Indeed, patient 1 had a family history of autoimmunity with her father having IgA nephropathy, which may represent an underlying predisposition to autoimmune disease. However, it is intriguing to consider the possibility that mitochondrial dysfunction may also promote a loss of immune tolerance, similar to that hypothesized in some models of rheumatoid arthritis pathophysiology.^20^ Additionally, it is possible that beta‐cell destruction secondary to mitochondrial dysfunction may lead to anti‐pancreatic antibody generation. Regardless, the severe presentation of diabetes mellitus in patient 1 emphasizes the importance of monitoring for diabetes in patients with mitochondrial disease from a young age.

Imaging findings in the patients in our cohort demonstrated initial confluent involvement of cerebral more than cerebellar white matter as well as progressive involvement of the brainstem and thalami. Interestingly, the basal ganglia were relatively spared in the imaging of patients 1 and 2. This contrasts with prior reports noting that the basal ganglia are sometimes the primary site of involvement in patients with NDUFS8‐related disorder,^8^ or more broadly in patients with complex I deficiencies.^21^ In the case of patient 2, the basal ganglia were ultimately involved as seen on autopsy, however, this was 7 months after initial imaging. However, our imaging findings are similar to cavitating leukoencephalopathies seen with mutations in other mitochondrial oxidative phosphorylation components.^22^ In addition, recent work suggested that in pediatric Leigh syndrome, nuclear DNA pathogenic variants are more likely than mitochondrial DNA pathogenic variants to have white matter involvement.^2^

In summary, we have presented three children from two unrelated families with an identical homozygous *NDUFS8* variant underlying a presentation of late infantile regression and progressive neurological decline. These variants are predicted to significantly disrupt *NDUFS8* function, corroborated by functional studies demonstrating decreased complex I activity. Patient 1's serial imaging demonstrates the severe progressive leukoencephalopathy that can be seen with disruptions of this gene, including involvement of the cerebellum and brainstem. We also provide an example of a severe presentation of new‐onset autoimmune diabetes in a patient with mitochondrial dysfunction, highlighting the importance of surveillance for diabetes in patients with mitochondrial disease.

## AUTHOR CONTRIBUTIONS

Siddharth Srivastava and Milena M. Andzelm were physicians who cared for patient 1 and drafted the original manuscript. Edward Yang assisted in critical interpretation of the radiological findings. John Christodoulou cared for patients 2 and 3 and John Christodoulou, Shanti Balasubramaniam and David R. Thorburn provided their clinical information and biochemical workup. Alison G. Compton provided mutational analysis. Kate Millington, Jia Zhu, Irina Anselm and Lance H. Rodan provided critical feedback of the manuscript and contributed to intellectual content. All authors have read/critically revised the manuscript.

## FUNDING INFORMATION

This work was supported by a Fellowship (David R. Thorburn) and grants (David R. Thorburn, John Christodoulou, Alison G. Compton) from the Australian National Health and Medical Research Council and the Victorian Government's Operational Infrastructure Support Program. The Chair in Genomic Medicine awarded to John Christodoulou is generously supported by The Royal Children's Hospital Foundation. We are grateful to the Crane and Perkins families for their generous financial support. Funding to Siddharth Srivastava is provided by the NIH NINDS (1K23NS119666‐01A1).

## CONFLICT OF INTEREST

Milena Andzelm, Shanti Balasubramaniam, Edward Yang, Alison Compton, Kate Millington, Jia Zhu, Irina Anselm, Lance Rodan, David Thorburn, John Christodoulou, and Siddharth Srivastava declare they have no conflicts of interest.

## ETHICS STATEMENT

All procedures followed were in accordance with the ethical standards of the responsible committee on human experimentation (institutional and national) and with the Helsinki Declaration of 1975, as revised in 2000. We have de‐identified patient data to the standards of the HIPAA Privacy rule and do not believe the information provided allows the identification of the patients through other means. Based on this, and because this is three or less cases, this does not meet the standards of human subjects research in accordance with the IRB standards of Boston Children's Hospital. This publication has also received ethics and governance approval from The Children's Hospital at Westmead.

## ANIMAL RIGHTS

This article does not contain any studies with animal subjects performed by any of the authors.

## Data Availability

This manuscript does not have associated supporting data.
